# Editorial: Impact of dietary nanoparticles on food allergy development and immune response

**DOI:** 10.3389/fimmu.2025.1693313

**Published:** 2025-09-22

**Authors:** Angela Rizzi, Sebastiano Gangemi

**Affiliations:** ^1^ UOSD Allergologia e Immunologia Clinica, Dipartimento Scienze Mediche e Chirurgiche, Fondazione Policlinico Universitario A. Gemelli IRCCS, Rome, Italy; ^2^ Department of Clinical and Experimental Medicine, School and Operative Unit of Allergy and Clinical Immunology, University of Messina, Messina, Italy

**Keywords:** food allergy, dietary nanoparticles, intestinal barrier, gut microbiota, *in vitro* and *in vivo* experiments

Food allergy is an inappropriate immune response to dietary antigens. It is a multifactorial condition with an increased incidence not only in industrialized countries but also in developing ones (1).

While the exact causes and pathogenic mechanisms of food allergies are still unclear, it is now widely accepted that the progression toward damage to the intestinal epithelial barrier is influenced by the intricate relationship between genetic susceptibility and environmental and immunological factors (2).

One such environmental factor is the unintentional exposure to inorganic dietary nanoparticles (IDNPs) ranging from 1 to 100 nanometers, such as titanium dioxide (TiO2NP or E171), zinc oxide (ZnONP), or silver (AgNP or E174) nanoparticles in everyday life, through the diet. All materials in the “nano” size present new and exciting chemical and physical properties compared to their conventional bulk counterparts that can be useful in several applications. For this reason, nanomaterials are used in a wide range of applications today, including agriculture, sustainable energy, the food chain, and medicine (3).

Although studies on the gastrointestinal tract are limited, it has been shown that nanoparticles can alter intestinal homeostasis and permeability by compromising the epithelial barrier and, in turn, activating the innate and adaptive immune response, possibly also affecting the gut microbiota. This could be relevant for patients with immunological diseases, such as those with inflammatory bowel disease (IBD) or food allergies (4, 5).

This Research Topic comprehensively describes several aspects of food allergies.

Starting with a report on the prevalence of this condition in Latin America, this Research Topic moves on to explore different pathogenetic mechanisms of damage underlying the genesis of food allergy: a translational animal model of α-Gal/red meat allergy based on gene deficiency and an *in vitro* and animal study of intestinal barrier impairment due to co-exposure to allergens and bacterial enterotoxins. An in-depth narrative review described the effects of exposure to inorganic nanoparticles on the intestinal barrier and their consequent potential pathogenic role in IBD. Readers will find four contributions to this Research Topic (three original articles and one review article) accessible here: www.frontiersin.org/research-topics/65925/impact-of-dietary-nanoparticles-on-food-allergy-development-and-immune-response (accessed on 21 August 2025).

Olaya-Hernández et al. conducted one of the few epidemiological studies on food allergies in Latin America. Due to its tropical and subtropical climate, high cultural diversity, and varied food preparation methods, Latin America is a region where food allergies are the most common cause of anaphylaxis (6, 7). For the first time, this study explored the prevalence and clinical characteristics of food allergies confirmed by an oral challenge in a cohort of 176 Colombians. The population was predominantly pediatric, with a mean age of two years. Surprisingly, no sensitization was found to tropical fruits but rather to eggs, shellfish, and cow’s milk. This was confirmed through skin tests and specific IgE. The positive rate for the oral challenge was 5.5% (only 12 patients): five patients with cow’s milk, five with shrimp and two with legumes.

Wang et al. chose the porcine model to better understand the pathogenic mechanisms underlying food allergies, and particularly α-Gal allergies, starting from the assumption that this animal reflects human intestinal physiology, anatomy and immune systems much more accurately than the murine model, which is already widely used by researchers (8). Previously, both peanut allergy (9, 10) and egg allergy (11) were investigated with the same animal model. To sensitize the animals to mimic tick bites in humans as closely as possible, the Authors preferred repeated intracutaneous injections of α-Gal rather than the “tape stripping” method, which mechanically compromises the porcine skin barrier with major stress to the animal. Consequently, these sensitized animals showed clear cutaneous and serum allergic responses with an increase in T helper 2 (Th2) cells, proallergic cytokines and epithelial-derived alarmins. Similarly, an increase in Th1 cells was observed, which are capable of triggering further inflammatory responses. Finally, re-exposure of pre-sensitized pigs to α-Gal also reproduced anaphylaxis with mediator release, fully confirming the validity of the animal model.

The *in vivo* and *in vitro* study conducted by Chinese researchers led by Yuan also focused on the same goal: a better understanding of the role of environmental factors such as bacterial enterotoxins in the development of food allergies. Interestingly, oral exposure to even low doses of *Staphylococcus aureus* enterotoxin B (SEB) in ovalbumin (OVA)-sensitized mice appeared to be a significant risk factor for the development and severity of food allergies. Indeed, histopathological analyses revealed that the intestinal barrier of mice simultaneously exposed to both OVA and SEB was more disrupted than that of the OVA group, with a loss of goblet cells, damaged epithelium, and overall tissue architecture distortion. Furthermore, real-time PCR analyses showed reduced expression of tight junction proteins (*claudin-2*, *Occludin*, and *ZO-1*) in the OVA + high SEB group, along with more Th2 polarization (more IL-13 and less IFN-γ) in mice co-exposed to low doses of SEB, again compared to the OVA-only group. Similarly, *in vitro* studies using cell co-culture models have shown that co-exposure to SEB and OVA plays a crucial role in the development of food allergies because it accelerates the activation of dendritic cells. This allows for increased allergen uptake and presentation by bone marrow dendritic cells to T cells, which upregulates genes involved in immune responses. Additionally, exposure to SEB causes a partial reduction in cecal flora biodiversity in OVA-sensitized mice.

Similar to food allergies, the prevalence of IBD has increased in industrialized countries alongside the increasing consumption of junk food and food additives containing various IDNPs. The closing study by Luo et al. exhaustively reviewed the role and the potential immunopathological mechanisms of these environmental factors in the development of IBD with a focus on the intestinal barrier in terms of its physical, chemical, biological, and immune barriers. Notoriously, the function of IDNPs in the body is quite intricate. While it is influenced by the shape, size, composition, surface properties, and state of aggregation of the nanoparticles themselves, the formation of a biological corona around the nanoparticles when they come into contact with various food ingredients or intrinsic components of the gastrointestinal tract (proteins, lipids, carbohydrates, etc.) can in turn alter the biocompatibility of the nanoparticles, altering their absorption and thus their toxicity. Although a growing body of literature has explored these aspects, research findings are often conflicting and lack definitive conclusions. This may be due to methodological differences, such as differences in the species, sex, age, and feeding habits of the animals used in the studies, or the intrinsic characteristics of the IDNPs chosen, or even to drug use, pre-existing conditions, or disease activity. Second, there are a limited number of *in vivo* studies on the chronic toxicity of IDNPs, although living beings are likely increasingly exposed to them over the long term. Similarly, there are no relevant studies investigating exposure levels and the effects of IDNPs in food packaging on humans without a unified standard for toxicity monitoring.

## Conclusion

We sincerely thank all the authors who submitted their articles and the reviewers who took the time to evaluate them. As an editorial team, we hope this Research Topic offers valuable insights for a better understanding of the epidemiological and pathophysiological aspects of immune-allergic diseases.
